# Nutritional status and the associated factors among people living with HIV: an evidence from cross-sectional survey in hospital based antiretroviral therapy site in Kathmandu, Nepal

**DOI:** 10.1186/s40795-020-00346-7

**Published:** 2020-06-15

**Authors:** Samip Khatri, Archana Amatya, Binjwala Shrestha

**Affiliations:** Central Department of Public Health, Institute of Medicine, Tribhuvan University, Maharajgunj, Kathmandu, Nepal

**Keywords:** Underweight, Anemia, Dietary diversity, People living with HIV, Body mass index

## Abstract

**Background:**

Nutritional status is the key concern among the people living with HIV but this issue has been failed to be prioritized in HIV strategic plan of Nepal. This study aims to assess the nutritional status among people living with HIV and determine their associated factors.

**Methods:**

A hospital based cross-sectional study was conducted where 350 people living with HIV attending the ART clinic were selected using systematic random sampling technique. Nutritional status among people living with HIV was assessed through anthropometry, body mass index; Underweight (body mass index < 18.5 kg/m^2^) and overweight/obesity (body mass index > 23 kg/m^2^). HIV related clinical factors such CD4 count, WHO stage, opportunistic infection, antiretroviral therapy regimen etc. were collected from the medical records. Socio-demographic data were collected using pretested structured questionnaire through interview technique. Multiple linear regression method was employed to determine the association between different independent factors and body mass index score.

**Results:**

The prevalence of underweight was found to be 18.3% (95% CI: 14.3–22.6). Most of the study participants were overweight/obese (39.1%). After subjection to multiple linear regression analysis, it was found that age, being male, being married, being in business occupation, smoking, hemoglobin level and antiretroviral therapy duration were significantly associated with body mass index score. Majority of the participants in our study lacked diversified food (62.3%).

**Conclusion:**

Overweight/obesity is an emerging problem among people living with HIV. This group of participants should be screened for the presence of non-communicable disease. This study also highlights the importance of nutritional program being an integral part of HIV/AIDS continuum of care. Therefore, an effort should be made to address the burden of malnutrition by addressing the identified determinants.

## Background

According to the global HIV statistics 2017, the number of people living with HIV around the world was about 36.9 million. Meanwhile, 21.7 million people living with HIV had access to ART, up from 17.1 million in 2015 and 8 million in 2010. As the result HIV/AIDS related deaths have fallen by 51% since the peak in 2004 [1]. In Nepal, the estimated number of people living with HIV as of December 2016 was 32, 735 [2], but lacks the information regarding their vulnerability to malnutrition.

The imbalance between the cellular supply of nutrient or energy and the body’s requirement for it to ensure proper growth, development and maintenance of specific functions results in malnutrition [3, 4]. Malnutrition is one of the major problems among those infected with HIV/AIDS. Various studies have shown that HIV infection, food insecurity and malnutrition are intricately linked to each other. In the absence of proper nutrition and treatment, HIV infection can lead to malnutrition which in turn impairs the immune system thereby progressing HIV to AIDS [5]. Underweight is a condition where macro and /or micronutrient supply are below the minimum dietary requirement which eventually leads to changes in body composition and diminished function [6, 7]. People living with HIV (PLHIV) with undernourishment manifests different conditions such as weight loss, muscle wasting, compromised immune system, micronutrient deficiency etc. and hence susceptible to the opportunistic infection [5].

National nutrition policy and strategy of Nepal although addresses the measures regarding nutrition such as breastfeeding by HIV positive mothers, it remains silent on the matter regarding the nutritional care of PLHIV. It does not consider PLHIV as nutritionally vulnerable group [8]. National demographic health survey provides data in the area of maternal and child health, nutrition and HIV/AIDS related knowledge and behavior. It is not particularly disaggregated in the matter regarding nutritional status of PLHIV [9]. The only hospital-based study conducted in 2013 in Nepal showed the prevalence of underweight among PLHIV to be about 20% [10]. Very few studies have been conducted to assess the nutritional status among PLHIV in context of our country. The prevalence of underweight is found to be quite different in similar studies done in various parts of world especially in the African country [11]. In one review paper, approximately 40–44% adult wasting and 59% of child malnutrition were reported [12].

A healthy balanced diet can improve the nutritional status of person with HIV/AIDS or delay the progression of HIV to AIDS but cannot cure or prevent the infection, though it helps to maintain normal body weight and fitness. Healthy balanced diet can optimize the immune system and thus improves the body’s ability to protect itself from the opportunistic infection [13]. Most of the PLHIV in resource limited settings are deprived from access to optimal quantities of well-balanced diet [11, 14]. Though the availability of highly effective antiretroviral therapy and Cotrimoxazole prophylaxis has potential to reduce the risk of morbidity, increase the life expectancy and halt the course of HIV infection; lack of adequate funding, treatment, care, support system and poor nutrition has contributed to higher morbidity and mortality of PLHIV in developing countries [15].

The Constitution of Nepal guarantees basic health services free of cost to Nepali citizens. As HIV control is one of the high-priority national development program, the National HIV Strategic Plan 2016–2021 carries the ethos of this constitutional provision to guarantee access to basic health services as a fundamental right of every citizen. Hence as a part of basic health service, ART services are provided free of cost to PLHIV which enables them to lead a healthy life (i.e. significant weight gain and increased CD4 count) which is possible only through viral suppression and adequate nutrient supplementation [16–20].

Manifestations of chronic HIV infection coupled with long term ART regimen include dyslipidemia, insulin resistance, cardiovascular disease, stroke and diabetes especially among those who are overweight [21–23]. Hence, it might be essential that those who are on higher risk of non-communicable disease should undergo dietary management [24]. An evidence from different study also suggests that undernourished PLHIV are 2–6 times more likely to die within the first 6 month of ART compared to those who have normal body mass index [25–29]. This supports the idea that nutritional component should be the core of any HIV intervention program in Nepal if any ART treatment is to be successful.

HIV infection is also frequently associated with hematologic abnormality especially anemia. Anemia is also an important contributor to morbidity and mortality among HIV infected individuals. In different study settings, about 60–80% of the PLHIV in their late stage are affected by anemia, making it most common hematologic manifestation [30].

The empirical evidence among PLHIV showed that being male [31], residing in the rural areas [32], household income less than two US dollar per day [33], illiteracy and college or university level education [10, 34] were significantly associated with underweight. Moreover, the presence of gastrointestinal symptoms [34, 35], opportunistic infections [34, 36], CD4 count [10], eating difficulty [31, 32, 37], ART status [31], current clinical condition [37], World Health Organization (WHO) stage [10, 34, 36], duration of ART [31, 37], nutritional support and dietary diversity [36], food security [10, 36, 38], and latrine availability [39] were reported as the determinants for underweight.

There is an urgent need for renewed focus on the use of resources for nutrition as a fundamental part of the comprehensive package of care at country level [31]. Recognizing the impact of nutrition on the progress of HIV/AIDS, the WHO has advocated that nutritional support to be integrated into national HIV/AIDS strategies [40]. But unfortunately, it has not been tangibly strategized in Nepal.

Our study aims to assess the nutritional status and its associated factors among people living with HIV visiting ART site in Kathmandu. The outcome of this study would be the evidence identifying the potential risk factors of underweight, and overweight/obesity as well among PLHIV.

## Methods

### Study design

A hospital based cross-sectional study design was used to assess the nutritional status and the associated factors.

### Setting of the study

According to National Centre for AIDS and STD Control 2017, there were total of 68 antiretroviral therapy sites throughout the country. Among these, 5 ART sites were present in Kathmandu district. Sukraraj Tropical and Infectious Disease Control hospital was purposively selected as a site for studying people living with HIV (PLHIV), as it is national referral hospital and receives patient from all over the Nepal for ART service and counselling. This can be comparatively more representative relative to other ART site. Study was done for the period of 6 month from July to December 2017.

### Study participants

ART register maintained at ART site of Sukraraj Tropical and Infectious Disease Control hospital was taken as sampling frame. Inclusion criteria for the study includes diagnosis of HIV at least 6 months prior to study period, age 18 years or more and has been under ART for at least a month. Those who were severely ill and pregnant women were excluded from the study.

### Sample size and sampling technique

The sample size was calculated using the formula; n’ = Z^2^ p (1-p)/d^2^ [41]. Previous study done shows the prevalence of underweight in PLHIV (*p* = 46.8%) [42]. At 5% level of significance [Z value at 5% level of significance: 1.96 (two tailed), absolute precision (d) = 5%], the sample size calculated was 382. Assuming the non-response rate of 5%, our final estimated sample size for the study (n) was 400. Systematic random sampling technique was employed to select the study participants. Our sample size (n’) was 382 and as per the patient flow record (ART register), average number of PLHIV visiting Sukraraj Tropical and Infectious Disease Control hospital per day was 60. So sampling fraction (K) = 382/60 i.e. 6.3 or 6. Therefore every 6th unit was selected.

### Data collection tools and procedures

Structured questionnaire was employed for data collection via face to face interview. The questionnaire was designed to capture socio-demographic, behavioral and anthropometric data. For BMI calculation, checking and recalibration of instrument was done for height and weight measurement. Mean of three measurement of each of the anthropometric parameter was taken and reported as final measurement. The body weight and height were measured by allowing the participant subject to light cloth and shoes taken off. Excess clothing like jacket, scarf etc was requested to remove during body weight measurement. For height measurement, participants were requested to stand erect, looking straight in the horizontal plane with feet together and knees straight. Blood sample was taken from the study participants who gave consent for the study in order to detect the hemoglobin level. Blood sample was drawn into vacutainer tube by experienced laboratory technician. Hemoglobin detection was done by using an instrument hematology analyzer (SLS method). HIV related clinical variables such as ART regimen, ART duration, CD4 count, WHO clinical staging, opportunistic infection was taken from the medical record of each of the participant. Data on these variables were collected during our study period i.e. at the time of data collection phase. The WHO clinical staging, obtained from the medical record was done by the chief consultant tropical medicine physician of the hospital. The economic status of the study participants was based on the Kuppuswamy’s socioeconomic scale [43]. Dietary diversity of the PLHIV was calculated using standardized individual dietary diversity score tool adopted from FAO guideline [44].

The questionnaire was first prepared in English and then translated to Nepali language. The tools were pretested on 25 PLHIV (about 10% of total study participants) at ART site of Bir hospital. After pretesting, cohabiting option was removed from marital status variable; education and occupation of the head of the family of study participants was included, food eating frequency in dietary diversity questionnaire was changed from 5 times per day [i.e. breakfast, lunch, afternoon (snack), evening (snack) and dinner] to 4 times per day [i.e. breakfast, lunch, afternoon (snack), and dinner]. Both the face and construct validity of the questionnaire was assured. Face validity was assessed during pretesting and modifications were made to make the question more understandable. Construct validity was ensured by making an accurate operational definition for each variable.

### Study definitions

One of the continuous variable in our study i.e. age has been categorized into three levels with equally spaced intervals i.e. 18–34 year, 35–51 year and 52–68 years of age. Other continuous variables such as BMI, hemoglobin level, ART duration, CD4 count and dietary diversity score has been categorized based on external criteria such as WHO guideline, national guideline/protocols/ manuals etc. Nutritional status, which is main outcome variable was based on the body mass index as per Asian BMI cut off limit [45, 46]. BMI was calculated as weight (kilogram) divided by height squared (meter), and categorized as underweight (BMI < 18.5 kg/m^2^), normal (BMI 18.5–22.9 kg/m^2^), overweight (BMI 23.0–27.4 kg/m^2^) and obese (BMI ≥ 27.5 kg/m^2^). Ethnicity was categorized as privileged (upper caste and relatively advantaged janajatis) and underprivileged (dalits, disadvantaged janajatis, disadvantaged non dalit terai caste and religious minorities). Hemoglobin status was categorized as anemic and non-anemic based on WHO guideline [47]. The dietary diversity score reflects the probability of micronutrient adequacy in the diet. The dietary diversity score was calculated by listing all the foods from among nine food groups (starchy staples, dark green leafy vegetables, vitamin A rich fruits and vegetables, other fruits and vegetables, organ meat, meat and fish, eggs, legumes, nuts and seeds and milk and milk products), that the study participants consumed over 24 h period be it outside or inside the home. The potential score range for individual dietary diversity is 0–9 and was calculated by summing the number of food groups consumed. Study participants received 1 point if they consumed food at least once during last 24 h period provided that food belonged to a particular food group and 0 point if they never consumed the food. The individual dietary diversity score (IDDS) in this study ranged from 1 to 7 with mean score i.e. mean (SD) being 4.22(1.04). The mean score was used as a cutoff point and those who consumed five and above food groups within 24 h period were considered as having high dietary diversity (diversified food) and those with IDDS below five i.e. consumed four and below food groups were considered as having low dietary diversity (undiversified food) [44]. ART regimen, which is the combination of three or more antiretroviral drugs for treating HIV infection was categorized as zidovudine and non-zidovudine based regimen. Zidovudine based regimen contains zidovudine drug among others in the combination.

Economic status of the study participants was assessed through Kuppuswamy socioeconomic scale which is based on the scoring of three variables i.e. education and occupation of the head of the family of study participants and monthly family income of study participants. This tool is applicable in context of India but can be used in case of Nepal through certain modification by taking into account the national price indices, which was 362.3 (2015/2016). Socio-economic status was categorized as lower (< 5), upper lower (5–10), lower middle (11–15), upper middle (16–25) and upper (26–29) [43, 48].

### Statistical analysis

Data collected from the study participants were entered into EpiData 3.1, with possible checks to avoid error. Data were imported into Statistical Package for Social Sciences (IBM statistics SPSS version 21.0 full version) and analysis was made further. Descriptive statistics was used to characterize the study participants regarding socio-demographic factors, HIV related clinical factors, dietary diversity and nutritional status using frequency, percentage, mean value (standard deviation) and median (range). All the continuous variables were tested for the normality before subjection to analysis (visual inspection of histogram, Kolmogorov-Smirnov test). Variables that were not normally distributed in our study (CD4 count, ART duration) were log transformed to approximate normal distribution. Dummy variables were created for those independent categorical variables that were categorized into more than two levels. The number of dummy variables created for a particular categorical variable is one less than the total number of response options for that categorical variable. A reference group for each of the categorical variable was decided before setting out for the dummy variable. All the diagnostic test regarding multiple linear regression assumptions i.e. absence of outliers, normality, linear relationship, absence of multicollinearity among independent variables was met. Univariate regression analysis of each independent variable was performed to test their degree of significant association with dependent variable (i.e. BMI score). Our multiple linear regression model was based on backward elimination technique with highest adjusted R^2^ value. The significant predictive capacity of the model developed was assessed using ANOVA statistics. Regression coefficient (beta weights) and 95% CI was used to estimate the magnitude of association. *P*-value less than 0.05 were considered significant.

### Ethical consideration

Written consent of participants was taken (before interview, for assessing their medical records and drawing their blood sample) and the objectives of the study were explained. Confidentiality and privacy were maintained properly. Approval letter was taken from hospital authority (Sukraraj Tropical and Infectious Disease Control Hospital) and Central Department of Public Health, Institute of Medicine to conduct the study. Ethical clearance was taken from Institutional Review Board, Institute of Medicine [Ref No. 83(6–11-E) 2/074/075].

## Results

### Total study participants in the analysis

Out of 400 estimated study population, only 350 were contacted and included in the study with the response rate being 87.5%. The data from the remaining 50 participants were not included in the analysis due to missing of the data points, declining to participate etc.

### Nutritional status of the study participants

Table 1 presents the proportion of study participants with different nutritional status. Our study revealed that only 18.3% of the study participant were undernourished with BMI being less than 18.5 kg/m^2^. Furthermore, nearly 39% of the study participants were overweight or obese. About 43% of the study participants had a normal BMI.

**Table 1:** Nutritional status of study participants (*N* = 350)

Nutritional status		N	Percentage	95% CI
Underweight	(BMI < 18.5)	64	18.3	14.3–22.6
Normal	(BMI 18.5–22.9)	149	42.6	37.7–48.0
Overweight	(BMI 23.0–27.4)	109	31.1	26.0–36.0
Obese	(BMI > 27.5)	28	8.0	5.2–11.1

### Socio-demographic characteristics

Table 2 depicts the socio-demographic characteristics of the study participants. Of the total sample analyzed, higher proportion (57.7%) of the study participants were from the age group 35–51 years. The mean age of the study participant was 38.9 years with standard deviation of 9.1 years and range from 18 to 67 years. Underweight was nearly two times higher among the study participants of age group 52–68 year (34.3%) compared to those of age group 18–34 year (16.8%) or 35–51 year (16.3%). Being overweight/obese was higher among 52–68 year age study participants (45.7%) compared to other study participants of different age group i.e. 18–34 year (37.2%) and 35–51 year (39.1%). Age of the study participants showed significant association with the nutritional status. Majority of the study participants were male (57.1%). Undernourishment was nearly as same in both male (18.5%) and female (18.0%). The prevalence of overweight/obesity was much higher in female (45.3%) as compared to that of male (34.5%). However, these differences were not significantly associated. Most of the study participant belonged to upper caste (45.4%), were married (69.1%), resided with family (87.1%) and belonged to upper lower socioeconomic class (56%). Married study participants showed highest prevalence of overweight/obesity (43.0%) and lowest prevalence of underweight (14.0%) compared to other groups. The relationship between the marital and nutritional status was significantly associated. About 27.7% of the study participants were deprived from schooling. With respect to the occupation, one in every four study participants were involved in some kind of household activities. The prevalence of overweight/obesity was higher among those who were involved in business (53.6%) but was lower among labor (25.5%). Similarly, the problem of undernourishment was highest among unemployed (33.3%) and lowest among farmers (7.9%). There were no differences in nutritional status among unemployed participants. Regarding the behavioral characteristic, it was found that most of the study participants avoided smoking (76.6%) and alcohol use (92%). About 23.4% of the study participants were involved in smoking cigarettes. Undernourishment was higher among cigarette smokers (26.8%) compared to non-smokers (15.7%), but being overweight/obesity was higher among non-smokers (43.7%) compared to smokers (24.4%). These differences in smoking habit showed significant association with nutritional status.

**Table 2:** Socio-demographic characteristics of the study participants by nutritional status (*N* = 350)

Variable	N (%)	Nutritional status			*p*-value
		Underweight *N* = 64	Normal *N* = 149	Overweight/Obese *N* = 137	
**Age Mean (SD)**	**38.9 (9.1)**				**0.03**
18–34 years	113 (32.3)	19 (16.8)	52 (46.0)	42 (37.2)	
35–51 years	202 (57.7)	33 (16.3)	90 (44.6)	79 (39.1)	
52–68 years	35 (10.0)	12 (34.3)	7 (20.0)	16 (45.7)	
**Sex**					**0.09**
Male	200 (57.1)	37 (18.5)	94 (47.0)	69 (34.5)	
Female	150 (42.9)	27 (18.0)	55 (36.7)	68 (45.3)	
**Ethnicity**					**0.90**
Upper caste	159 (45.4)	28 (17.6)	70 (44.0)	61 (38.4)	
Disadvantaged janajatis	95 (27.1)	18 (18.9)	40 (42.1)	37 (38.9)	
Relatively advantaged janajatis	61 (17.4)	11 (18.0)	23 (37.7)	27 (44.3)	
Dalits	20 (5.7)	6 (30.0)	8 (40.0)	6 (30.0)	
Disadvantaged non dalit terai caste	13 (3.7)	1 (7.7)	7 (53.8)	5 (38.5)	
Religious minorities	2 (0.6)	0 (0.0)	1 (50.0)	1 (50.0)	
**Marital status**					**0.01**
Married	242 (69.1)	34 (14.0)	104 (43.0)	104 (43.0)	
Widowed	53 (15.1)	13 (24.5)	22 (41.5)	18 (34.0)	
Unmarried	34 (9.7)	11 (32.4)	17 (50.0)	6 (17.6)	
Separated/Divorced	21 (6.0)	6 (28.6)	6 (28.6)	9 (42.9)	
**Current residence**					**0.47**
With family	305 (87.1)	54 (17.7)	127 (41.6)	124 (40.7)	
Single	31 (8.9)	5 (16.1)	16 (51.6)	10 (32.3)	
Organization	12 (3.4)	4 (33.3)	5 (41.7)	3 (25.0)	
With friends	2 (0.6)	1 (50.0)	1 (50.0)	0 (0.0)	
**Education**					**0.59**
No schooling	97 (27.7)	19 (19.6)	40 (41.2)	38 (39.2)	
Primary	59 (16.9)	11 (18.6)	23 (39.0)	25 (42.4)	
Lower secondary	69 (19.7)	11 (15.9)	37 (53.6)	21 (30.4)	
Secondary/Higher Secondary	109 (31.1)	20 (18.3)	45 (41.3)	44 (40.4)	
Graduate/Post-graduate	16 (4.6)	3 (18.8)	4 (25.0)	9 (56.3)	
**Occupation**					**0.01**
Household work	87 (24.9)	19 (21.8)	28 (32.2)	40 (46.0)	
Employee at private	74 (21.1)	18 (24.3)	32 (43.2)	24 (32.4)	
Labor	71 (20.3)	12 (16.9)	41 (57.7)	18 (25.4)	
Business	56 (16.0)	6 (10.7)	20 (35.7)	30 (53.6)	
Agriculture	38 (10.9)	3 (7.9)	20 (52.6)	15 (39.5)	
Government employee	12 (3.4)	2 (16.7)	4 (33.3)	6 (50.0)	
Unemployed	12 (3.4)	4 (33.3)	4 (33.3)	4 (33.3)	
**Economic status**					**0.12**
Upper middle	61 (17.4)	11 (18.0)	21 (34.4)	29 (47.5)	
Lower middle	89 (25.4)	15 (16.9)	35 (39.3)	39 (43.8)	
Upper lower	196 (56.0)	37 (18.9)	93 (47.4)	66 (33.7)	
Lower	4 (1.1)	1 (25.0)	0 (0.0)	3 (75.0)	
**Smoking status**					**0.04**
Yes	82 (23.4)	22 (26.8)	40 (48.8)	20 (24.4)	
No	268 (76.6)	42 (15.7)	109 (40.7)	117 (43.7)	
**Alcohol drinking status**					**0.50**
Yes	28 (8.0)	6 (21.4)	9 (32.1	13 (46.4)	
No	322 (92.0)	58 (18.0)	140 (43.5)	124 (38.5)	

### HIV related clinical characteristics of study participants

Nearly 39% of the study participant were having Zidovudine based ART regimen at the time of study. Study participant who were receiving ART for 2 years or more (i.e. 24 month or more) were about 67%. The median duration of ART use in this study was 48 months (range: 6–168 month). Majority of the study participant did not have opportunistic infection (96.6%). Most of the study participants were in WHO stage I accounting for about 43.7%. The median CD4 count of the study participants was 390 cells/mm^3^ (range: 5–1713). About half of the study participants (50.3%) had CD4 count between 200 and 500 cells/mm^3^. Overweight/obese participants in our study tended to have higher CD4 count compared to others, however the differences were not significantly associated. About 32% of the participants were anemic in our study. The mean hemoglobin level of the male participants was 13.7 g/dl (range: 8.2–18.2) whereas that of female participants was 12.4 g/dl (range: 8.1–17.0). The prevalence of underweight among the anemic participants was 23.2% whereas that of overweight/obese, among nonanemic participants was 45.4%. These differences observed were significantly associated (Table 3).

**Table 3:** HIV related clinical characteristics of the study participants by nutritional status (*N* = 350)

Variable	N (%)	Nutritional status			*p* value
		Underweight N = 64	Normal N = 149	Overweight/Obese N = 137	
**ART regimen**					**0.17**
Non-zidovudine based regimen	215 (61.4)	39 (18.1)	84 (39.1)	92 (42.8)	
Zidovudine based regimen	135 (38.6)	25 (18.5)	65 (48.1)	45 (33.3)	
**ART duration Median (range)**	**48 (6, 168)**				**0.12**
≥ 24 months	234 (66.9)	49 (20.9)	100 (42.7)	85 (36.3)	
< 24 months	116 (33.1)	15 (12.9)	49 (42.2)	52 (44.8)	
**Opportunistic infection**					**0.15**
No	338 (96.6)	61 (18.0)	143 (42.3)	134 (39.6)	
HCV	7 (2.0)	0 (0.0)	5 (71.4)	2 (28.6)	
Tuberculosis	4 (1.1)	2 (50.0)	1 (25.0)	1 (25.0)	
Syphilis	1 (0.3)	1 (100.0)	0 (0.0)	0 (0.0)	
**WHO stage**					**0.04**
Stage I	153 (43.7)	21 (13.7)	64 (41.8)	68 (44.4)	
Stage II	81 (23.1)	25 (30.9)	32 (39.5)	24 (29.6)	
Stage III	80 (22.9)	13 (16.3)	36 (45.0)	31 (38.8)	
Stage IV	36 (10.3)	5 (13.9)	17 (47.2)	14 (38.9)	
**CD4 count Median (range)**		**368.5 (13, 1713)**	**382 (5, 1468)**	**440 (21, 1695)**	**0.69**
Less than 200 cells/mm^3^	51 (14.6)	12 (23.5)	20 (39.2)	19 (37.3)	
Between 200 to 500 cells/mm^3^	176 (50.3)	34 (19.3)	75 (42.6)	67 (38.1)	
Greater than 500 cells/ mm^3^	123 (35.1)	18 (14.6)	54 (43.9)	51 (41.5)	
**Hemoglobin level**					**0.02**
Non-anemic	238 (68)	38 (16.0)	92 (38.7)	108 (45.4)	
Anemic	112 (32)	26 (23.2)	57 (50.9)	29 (25.9)	

### Dietary diversity pattern

The individual dietary diversity score (IDDS) ranged from 1 to 7 with cutoff, mean dietary diversity score being 4.22 (1.04). About 62.3% of study participants lacked diversified food (Table 4). About 44.7% of the study participants who consumed high diversified food were overweight/obese. Majority of the study participants consumed diet from the food group such as starchy staple (100.0%), other fruits and vegetables (78.9%), legumes, nuts and seeds (87.1%). About half or more of the study participants lacked micronutrient rich food groups in their diet such as organ meat (99.1%), dark green leafy vegetables (50.6%), Vitamin A rich fruits and vegetables (92.9%), eggs (79.7%), milk and milk products (77.1%). About 42.8% of the study participants who consumed dark green leafy vegetables were overweight/obese. Undernourishment was lower (20.3%) among the study participants who lacked dark green leafy vegetables in their diet. However, the relationship was not statistically significant. Most of the study participants (60%) who consumed Vitamin A rich fruits and vegetables had a normal BMI but the association was not significant. Consumption of organ meat did not contribute significantly to the nutritional status of the study participants. The prevalence of underweight (18.3%) was same in both the study participants i.e. those who consumed and did not consume egg. About 47.5% of the study participants who consumed milk and milk products were overweight/obese.

**Table 4:** Dietary diversity and dietary pattern of the study participants (N = 350)

Variable	N (%)	Nutritional status			*p* value
		Underweight *N* = 64	Normal *N* = 149	Overweight/Obese *N* = 137	
**Dietary diversity** Mean (SD)	4.22 (1.04)				**0.25**
Low	218 (62.3)	42 (19.3)	98 (45.0)	78 (35.8)	
High	132 (37.7)	22 (16.7)	51 (38.6)	59 (44.7)	
**Starchy staple**					**–**
Yes	350 (100.0)	64 (18.3)	149 (42.6)	137 (39.1)	
No	0 (0.0)	0 (0.0)	0 (0.0)	0 (0.0)	
**Dark green leafy vegetables**					**0.33**
Yes	173 (49.4)	28 (16.2)	71 (41.0)	74 (42.8)	
No	177 (50.6)	36 (20.3)	78 (44.1)	63 (35.6)	
**Vitamin A rich fruits and vegetables**					**0.18**
Yes	25 (7.1)	3 (12.0)	15 (60.0)	7 (28.0)	
No	325 (92.9)	61 (18.8)	134 (41.2)	130 (40.0)	
**Other fruits and vegetables**					**0.37**
Yes	275 (78.9)	53 (19.3)	112 (40.7)	110 (40.0)	
No	75 (21.4)	11 (14.7)	37 (49.3)	27 (36.0)	
**Organ meats**					**0.82**
**Yes**	3 (0.9)	1 (33.3)	1 (33.3)	1 (33.3)	
No	347 (99.1)	63 (18.2)	148 (42.7)	136 (39.2)	
**Meat and fish**					**0.89**
Yes	197 (56.3)	37 (18.8)	85 (43.1)	75 (38.1)	
No	153 (43.7)	27 (17.6)	64 (41.8)	62 (40.5)	
**Eggs**					**0.63**
Yes	71 (20.3)	13 (18.3)	27 (38.0)	31 (43.7)	
No	279 (79.7)	51 (18.3)	122 (43.7)	106 (38.0)	
**Legumes, nuts and seeds**					**0.80**
Yes	305 (87.1)	57 (18.7)	128 (42.0)	120 (39.3)	
No	45 (12.9)	7 (15.6)	21 (46.7)	17 (37.8)	
**Milk and milk products**					**0.21**
Yes	80 (22.9)	12 (15.0)	30 (37.5)	38 (47.5)	
No	270 (77.1)	52 (19.3)	119 (44.1)	99 (36.7)	

### Analysis of association

All the independent variables were analyzed for their association with BMI score. The result of univariate linear regression analysis showed that being male, marital status i.e. both married and unmarried, occupation i.e. business and employee at private, cigarette smoking, WHO II stage, hemoglobin (Hb) level and ART duration were significantly associated with BMI score (data not presented in table). We conducted multiple linear regression analysis using backward elimination technique. Among different model obtained, we selected the model with highest adjusted R^2^ value. The regression model showed that age, hemoglobin level, ART duration, being male, being married, being in business occupation and smoking significantly associated with BMI score (Table 5).

**Table 5:** Multiple linear regression analysis with BMI score

Independent Variable	β	Standardized β	*p*-value	T	95% CI for β
Constant	14.77	–	< 0.001	7.93	11.10–18.43
Age	0.05	0.13	**0.018**	2.39	0.01–0.09
Hemoglobin level	0.36	0.17	**0.002**	3.09	0.13–0.59
ART duration	−0.02	−0.16	**0.004**	−2.91	−0.03-(−0.01)
CD4 count	0.01	0.07	0.202	1.28	−0.001-0.002
Dietary diversity score	0.29	0.08	0.149	1.45	−0.10-0.68
Male sex	−1.28	−0.15	**0.012**	−2.53	−2.16-(−0.27)
Privileged Ethnicity	−0.59	−0.07	0.162	−1.40	−1.42-0.24
Married	1.27	0.15	**0.004**	2.90	0.41–2.12
Business	1.15	0.11	**0.035**	2.11	0.08–2.22
Smoking	−1.63	−0.18	**0.002**	−3.16	−2.64-(− 0.61)
Tuberculosis as opportunistic infection	−2.57	−0.07	0.172	−1.37	−6.26-1.12
WHO II stage	−0.72	− 0.08	0.136	−1.50	− 1.66-0.23

R = 0.401, R^2^ = 0.161, Adjusted R^2^ = 0.131,  F (12, 337) =5.382, *p* < 0.001

With regard to multiple linear regression model, the estimated BMI of the study participants changed by 0.05 kg/m^2^ for every additional year in the age, holding all other variable constant. Male study participants had lower BMI by approximately 1.2 kg/m^2^ holding another variable constant. Similarly, smoking study participants had lower BMI by almost 1.63 kg/m^2^. Married participants had higher BMI by almost 1.27 kg/m^2^ compared to those who were single after marriage i.e. divorced/widowed/separated. Similarly, study participants who were involved in business showed higher BMI by 1.15 kg/m^2^ compared to those who were unemployed.

The estimated BMI of the study participants lowered by almost 0.02 kg/m^2^ for each additional month of ART regimen holding another variable constant. Similarly, the estimated change in the BMI of the study participants rose by almost 0.36 kg/m^2^ for each additional unit of hemoglobin level. Other independent variable such as CD4 count, dietary diversity score, privileged ethnicity, WHO II stage and tuberculosis as opportunistic infection in the model was not significantly associated but still contributed to the overall variation of the BMI score.

The coefficient of determination (R^2^) was found to be 0.161 i.e. the model’s degree of explaining the variance in the BMI score was 16.1%. According to the ANOVA statistics [F (12,337) =5.382, *p* < 0.05] the independent variables in the model were significantly predictive of BMI score.

## Discussion

Our study estimated the proportion of nutritional status among PLHIV visiting a specific ART site in Kathmandu and determines the association between various factors and BMI score. Nutrition and HIV are strongly related and complement each other [42]. The body of knowledge showing the relationship between nutritional status and HIV infection pertaining to context of Nepal is very limited. The only single study conducted previously in 2013 Kathmandu, Nepal showed associated factors of nutritional status and the quality of life among PLHIV [10]. Our study in addition to assessing the nutritional status looks into other important factors such as dietary diversity and anemia.

### Nutritional status, anemia and dietary diversity among PLHIV

The role of HIV infection has been well documented with wasting as one of the most visible signs of malnutrition in patient who progress to AIDS [7]. Our study found out that about 18.3% of PLHIV were undernourished. The finding was comparatively lower than the previous study conducted in Nepal where the prevalence of underweight was nearly 20% [10]. Different studies show wide range of undernourishment among PLHIV, where it can be as low as 10.3% [49] and as high as 46.8% [42]. The difference might be due different socio-cultural, political, economic factors of the countries and also the health care system’s sensitivity on addressing the prevention, treatment, care and support of PLHIV. The decreased prevalence of underweight among PLHIV in our study compared to previous study conducted in Kathmandu, Nepal might be due to various factors such as increased coverage of ART services, CD4 count and viral load testing services throughout the country. In addition to these, the provision of community and home-based care, management of co-infection under HIV treatment, care and support services might indirectly contribute to the nutritional wellbeing of the PLHIV.

Since much of the study done in developing countries looks into undernourishment, with underlying assumption that HIV infection is usually associated with weight loss [50], this study also looks into the proportion of PLHIV who are overweight or obese (39%). Obesity and overweight, which are the major risk factors for the non-communicable disease such as diabetes, cardiovascular disease, hypertension and cancer in the general population, are increasingly affecting PLHIV [51]. There have been substantial studies in developed countries that PLHIV on ART treatment experience increase in body mass index but their impact in terms of being overweight and obese is unknown [50].

Anemia is one of the most common hematologic abnormality in the patient with HIV and has been associated with disease progression and poor clinical outcomes [52]. The overall prevalence of anemia in our study was found to be 32% as per the hemoglobin cutoff value given by WHO [47]. The finding was very low as compared to the study done previously in Tribhuvan University Teaching Hospital (42%) [53] and as well as community-based study done in Nepal in 2014 showing 55.8% [54]. The cause of lower prevalence of anemia in our study might be multifactorial such as frequent monitoring of anemia in ART site, switching from zidovudine-based regimen, lower prevalence of opportunistic infection or better nutritional care etc.

Regarding dietary factors, our study revealed that more than half of the people living with HIV had low dietary diversity (62.3%). Most of the study done in Africa shows the similar trend with dietary diversity being low in about 59% of the PLHIV [55]. One of the reasons for low dietary diversity might be associated with poor dietary habits and poor household food security status of PLHIV. All the participants in our study consumed carbohydrate rich starchy staple such as rice but lacked micronutrient rich food such as organ meat, green leafy vegetables, Vitamin A rich foods, eggs and milk. This might also be due to the fact that most of the study participants belonged to lower economic class (57.1% i.e. both lower and upper lower) so could not afford micronutrient rich foods. The dietary habits of the people in developing countries are based on monotonous, energy dense and poor micronutrient source of starchy staples [56].

### Factors associated with BMI score

Various factors ranging from socio-demographic characteristics, HIV related clinical factors and dietary patterns plays an important role in determining the nutritional status (BMI score) of people living with HIV. Our study showed that age, hemoglobin level, ART duration, being male, being married, involved in business as occupation and smoking were potential factors that influenced the BMI score.

The significant variable in the model i.e. smoking, being male and ART duration was associated with the decrease in the BMI of the study participants. One of the reasons for the lower BMI among smoker might be due to the fact that smoking increases the chance of opportunistic infection, as it suppresses the host defenses and alters respiratory environment and thereby indirectly contributing to malnutrition through bacterial pneumonia. Furthermore, smoking impedes the long-term quality of life in this group of population. The inverse relationship between cigarette smoking and BMI may also be explained by the biological function of nicotine. Nicotine during cigarette smoking acutely increases energy expenditure and reduces appetite which could explain the lower body weight found in smokers [57].

The lower BMI among male study participants in our study might be due to their smoking habit compared to female (38% Vs 4%; data not shown in table). Beside this, various studies also suggest that males are likely to be undernourished compared to female because of the fact that they present late for HIV treatment and care with advanced HIV disease and low CD4 counts. Lower CD4 counts has been associated with moderate to severe malnutrition [58].

Although there is greater tendency of increasing BMI after initiation of ART, our study showed the inverse relationship between ART duration and the BMI score. The findings from different study have shown that the relationship between ART duration and BMI is inconsistent [59–61]. The differences in the ART duration may account for the variations in the relationship between ART duration and BMI [59]. One of the explanations for the lower BMI might be associated with symptoms such as difficulty in breathing, nausea, vomiting and oral infections which generally occurs during initial month after initiation of ART. Oral infection limits the ability to chew and swallow food leading to loss of appetite and hence reduced calorie intake. Additionally, poor adherence to ART or drug failure results in disease progression which in turn leads to weight loss [59]. However, our study did not evaluate these symptoms or drug adherence.

Being married and in business occupation was positively associated with BMI score. Married study participants had higher BMI compared to divorced/widowed/separated. The chances of undernourishment (lower BMI) are higher among those with marital disruption. Marriage may be associated with better nutrition in variety of ways. The earning potential of both the partner might improve their economic wellbeing, which in turn may improve their nutritional status. In addition, spouse may play an important role in monitoring and encouraging healthy behaviors (such as good eating habits and regular exercise), as well as in discouraging unhealthy ones (such as smoking or heavy drinking). Those who were involved in business showed higher BMI compared to those who were unemployed. One of the reasons for higher BMI among those with business occupation might be associated with their higher income which in turn increases their ability to spend money for food. But it is also true that this group of study participants due to their sedentary lifestyle and lack of physical activities are far more prone to develop overweight/obesity.

The study also showed the positive association between hemoglobin level and BMI. The trend analysis from various studies explains the fact that increase in the hemoglobin level are associated with increase in CD4 count. Higher CD4 count improves immune reconstitution, slowers disease progression, thereby improving the BMI of the study participants [62]. The longitudinal study design is necessary to see the effect of HIV related parameters on malnutrition or vice-versa since the nutritional status could modulate the immunological response to HIV infection over time [63].

The cross-sectional nature of the study limits the information regarding the effect of malnutrition on PLHIV and also the cause of anemia in PLHIV. One of the limitations of our study is the establishment of temporality with malnutrition. The possible association may not equate with causal relationship that may exist with BMI score. The final model’s degree of explaining variance i.e. coefficient of determination (R^2^) of BMI score is still relatively low, which is one the major limitation of the study. BMI has been noted for its rough reflection of body mass and does not measure the nutritional intake history and metabolic changes due to diet related problem. The dietary diversity score does not indicate the quantity of food consumed. Enough probing was done to avoid possible recall bias in the study participants as they had to recall on the diet, consumed over 24h period. The possibility of measurement bias was avoided using well calibrated instrument and taking mean value of three measurements for each of the anthropometric parameter. The result of our study is based on relatively small, single centered, urban survey in Kathmandu. Considering the fact that different region of Nepal varies in socio-demographic structure and dietary habits, more large scale and multicenter study is required for further confirmation of our findings.

National HIV strategic plan 2016–2021 advocates food and nutrition intervention being a part of package of care, treatment and support services for people living with HIV [2], but unfortunately it has not yet been effectively implemented because of low priority and limitation of budget required for the nutritional survey of PLHIV at the national level. This study could serve as the baseline for the large scale study on nutrition and HIV/AIDS. It also encourages healthcare providers to strengthen the nutritional assessment as a part of continuum of care particularly on the light of the fact that antiretroviral therapy becomes effective when there is proper nutritional intervention.

## Conclusion

The result of this study provides data on the characteristics of nutritional status of PLHIV and the factors that are significantly associated with BMI score. In our study, the prevalence of overweight/obesity was found higher among the study participants compared to being underweight. Overweight/obesity has become an emerging problem among PLHIV which is the major risk factor for non-communicable diseases (NCDs). Hence there is an urgent need to integrate the screening program for NCDs into HIV treatment and care services before any further complication arises. Male participants who smoke cigarette are at higher risk of being underweight, hence they particularly need to reduce their smoking habit. PLHIV who are in business occupation particularly needs have a balanced diet and involve in regular physical exercise as they are more vulnerable to develop overweight/obesity. Married participants are protected group however the divorced/separated participants are at higher risk of being underweight. Effort should be made to encourage the PLHIV to consume diversified food.

## Data Availability

The datasets used in the study are not available publicly but can be made available upon the reasonable request and with permission of Institute of Medicine and Sukraraj Tropical and Infectious Disease Control Hospital.

## Abbreviations

AIDS: Acquired Immunodeficiency Syndrome
ART: Antiretroviral Therapy
BMI: Body Mass Index
CD4: Clustered Determinant 4
CI: Confidence Interval
HIV: Human Immunodeficiency Virus
NCDs: Non-communicable Diseases
PLHIV: People Living with Human Immunodeficiency Virus
SLS: Sodium Laurate Sulphate
SPSS: Statistical Package for Social Science
TUTH: Tribhuvan University Teaching Hospital
WHO: World Health Organization
